# Prevalence of Hepatitis B Carrier Status and Its Negative Association with Hypertensive Disorders in Pregnancy

**DOI:** 10.1155/2021/9912743

**Published:** 2021-10-13

**Authors:** W. Y. Lok, C. W. Kong, W. W. K. To

**Affiliations:** Department of Obstetrics and Gynaecology, United Christian Hospital, Kwun Tong, Hong Kong

## Abstract

**Results:**

In a total cohort of 87889 deliveries over a period of 20 years, the prevalence rate of HBV fell from around 10-11% to around 6-7% in the last 5 years of the study. A negative association between chronic HBV carrier status and all gestational hypertensive disorders could be demonstrated. An apparent protective effect of HBV carrier status was apparently more robust against preeclampsia than gestational hypertension, as the negative association with preeclampsia was consistently observed throughout the study period. A logistic regression model showed that advanced maternal age, multiple pregnancies, obesity, and significant medical disorders were positively correlated with gestational hypertensive disorders, while multiparity and positive HBV carrier status were negatively correlated.

**Conclusion:**

Chronic HBV carrier status appeared to have a protective effect against the development of preeclampsia and gestational hypertension in an endemic area with high HBV prevalence rates.

## 1. Introduction

Hepatitis B (HBV) is a common cause of chronic liver disease worldwide and with particularly high prevalence rates in south-east Asia [1]. Maternal chronic HBV status has been associated with various adverse pregnancy outcomes including gestational diabetes mellitus, postpartum haemorrhage, intrahepatic cholestasis, preterm deliveries, caesarean section, and small for gestational age [2–4]. The reported relationship between chronic HBV virus infection on the development of gestational hypertension and preeclampsia in the literature had been conflicting and controversial and varied from a positive association [5, 6] to no association [3, 7, 8] to a negative association [9–11]. A recent meta-analysis of published studies up to early 2016 concluded that chronic HBV infection is associated with decreased risk of preeclampsia [12]. However, it could be seen that the principal studies that contributed most to the conclusion of a negative association consisted of large cohorts in which the prevalence of chronic HBV carrier status was around 10% of the total obstetric population. A previous study from Hong Kong reported a negative association between chronic HBV carrier status and gestational hypertension based on a retrospective cohort of 13,946 patients delivered between 1997 and 2000 [11]. Hong Kong has all along been endemic for chronic HBV infection, and similar to the rest of China, the prevalence of HBV carrier status has for many years been hovering around 10% [1] but decreasing to around 5-6% in recent years [13]. The aim of this study, therefore, was to evaluate whether such negative association with hypertensive disorders in pregnancy could continue to be demonstrated with the falling prevalence of chronic hepatitis carrier status. While the primary outcome will be the overall association between the prevalence of chronic HBV carrier status and hypertensive disorders in pregnancy as well as other significant pregnancy complications, the data yielded will verify whether such associations will be influenced by the changing prevalence of chronic HBV carrier status in the obstetric population.

## 2. Methods

A retrospective analysis of all patients who delivered in a tertiary referral obstetric unit, United Christian Hospital, Hong Kong, over a 20-year period from 2000 to 2019 was carried out using a comprehensive obstetric database. The unit served a local population of around 0.55 million at the start of the study period but increased to 0.75 million over the 18 years, with an average annual delivery rate of around 4400. Women with delivery after 24 weeks of gestation were included in the present study. All were screened for HBV surface antigen (HbsAg) at the time of booking or first presentation using a qualitative enzyme-linked immunosorbent assay (ELISA), and a positive test was defined as positive carrier status. Additional investigations including liver function tests and screening for hepatitis e antigen or hepatitis c (HCV) status were performed as clinically indicated. HBV viral DNA assays were not routinely performed, nor any protocol for antenatal antiviral treatment to decrease maternal-fetal transmission during the study period. Women who were diagnosed to have active hepatitis or an acute flare of chronic hepatitis for any cause during the pregnancy were excluded in the analysis. As part of our routine protocol, in addition to universal HBV vaccination at birth, all babies born from HBV positive mothers were given passive immunization with immunoglobulins as well.

The obstetric management of women diagnosed with gestational hypertension and preeclampsia followed established authoritative guidelines and departmental protocols. The categorization of the diagnoses of hypertensive disorders of pregnancy were in accordance with the ICD-9 coding definitions for preexisting hypertension, gestational hypertension, and preeclampsia and eclampsia, such that ***gestational hypertension*** would include all women with pregnancy related hypertension without proteinuria, ***preeclampsia*** would include all preeclampsia and eclampsia, and ***all gestational hypertensive disorders*** would include the sum of both gestational hypertension and preeclampsia groups. Additional key epidemiological parameters including age, parity, body mass index, incidence of various pregnancy complications, and immediate pregnancy outcome were extracted. The data were also stratified into four 5-year intervals to specifically evaluate any association between HBV status and hypertensive disorders.

The study was approved by the Research Ethics Committee of Kowloon East Cluster, Hospital Authority, Hong Kong. The Statistical Package for Social Sciences for Windows package version 23 was used for data entry and analysis. Descriptive categorical data were expressed as numbers and percentages and compared and analyzed by the chi-squared test where appropriate. A logistic regression model was constructed to evaluate the independent risk factors associated with preeclampsia and all gestational hypertensive disorders, and a *p* value of <0.05 is considered statistically significant.

## 3. Results

Of all eligible deliveries during the study period, 24 (0.3%) were excluded due to acute hepatitis or other active liver diseases, so that 87889 were included in the final analysis. The annual delivery rate fluctuated from 3440 to 5552, while the prevalence of HBV carrier status gradually dropped from 10-11% in the first decade to 5.3% in 2019 (Table 1), giving an overall HBV positive rate was 9.56%. There were slightly more non-Chinese women in the HBV positive group, the most being Filipinos and Pakistanis. There were no significant differences in the proportion with advanced maternal age (>35), parity, and various antenatal complications including gestational diabetes mellitus, cardiac, renal, and respiratory or neurological diseases between chronic HBV carriers and controls, nor were there any differences in the immediate perinatal outcome and the mode of delivery between HBV carriers and controls. Of the HBV carriers, 44 (0.5%) were on maintenance therapy with tenofovir or other antiviral treatment and were classified as having HBV-related liver diseases. Of the HBV-negative women, more had thyroid disorders including Graves disease or autoimmune thyroiditis (*p*=0.029) and more had induction of labour (*p* < 0.001) and small-for-gestational-age babies (*p*=0.018) (Table 2).

When the data were categorized into four 5-year intervals, it could be observed that the prevalence of chronic HBV carrier status was consistently high in the first 15 years, being 10.2%, 11.05, and 9.7% for the first 15 years, but dropped to 6.8% between 2014 and 2019. When the relationship between chronic HBV carrier status and hypertensive disorders was evaluated in each of these 5-year intervals, a negative association was consistently seen with preeclampsia/eclampsia and total hypertensive disorders, while the association for gestational hypertension was seen in the first decade but became no longer significant in the latter two 5-year intervals (Table 3).

Univariate analysis showed that parity, advanced maternal age, multiple pregnancies, and significant medical disorders that could predispose to gestational hypertensive disorders (including chronic hypertension, chronic renal diseases, preexisting or gestational diabetes mellitus, and autoimmune disorders such as systemic lupus erythematosus) were significantly related to preeclampsia and all gestational hypertensive disorders (Table 4). While there were fewer smoking women in the hypertensive groups, the differences were not statistically significant, probably because the overall incidence of smoking in the cohort was very low (around 2%). A logistic regression model using the presence or absence of gestational hypertension and preeclampsia/eclampsia, respectively, as the dependent outcome against the six epidemiological risk factors that were significant on univariate analysis showed that advanced maternal age (odds ratio (OR) 1.25 for all gestational hypertensive disorders and 1.36 for preeclampsia/eclampsia, respectively); multiple pregnancies (OR 1.97/2.09); obesity (OR 1.10/1.31); and significant medical disorders (OR 1.75/3.13) were consistently positively correlated, while multiparity (OR 0.78/0.79) and positive HBV status (OR 0.68 and 0.59) were negatively correlated to all gestational hypertensive disorders and preeclampsia (Table 5).

## 4. Discussion

The current findings consistently demonstrated a negative association between chronic HBV carrier status and the development of gestational hypertension and preeclampsia/eclampsia, respectively, as well as with all hypertensive disorders, both when the 20-year data were analyzed in total, or after stratifying into 5-year intervals. The purpose of dividing the data into five-year intervals was to allow any trends related to the incidence of chronic HBV to be more readily observable over a longer interval by mitigating the variable year to year fluctuations. The logistic regression model showed that HBV status was an apparently independent negative risk factor for gestational hypertensive disorders.

The prevalence of chronic hepatitis B carrier status in Hong Kong has all along been high at around 10% or more until recent years when the hepatitis B vaccination introduced in the eighties started to take its effects in the female reproductive population [13]. Similar trends with a gradually falling prevalence have also been shown in other endemic areas such as mainland China [1]. The protective effect of chronic HBV carrier status against hypertensive disorders was apparently more robust against preeclampsia than against gestational hypertension, as the negative association was no longer statistically significant for latter category in the 2010–2014 and 2015–2019 intervals when the prevalence of HBV was also gradually dropping. The incidence of superimposed preeclampsia and eclampsia were too low to show a significant difference between those who were HBV carriers and those who were not, despite analyzing the data in cumulative 5-year intervals. However, the modestly higher incidence of labour induction and small-for-gestational-age fetuses in the non-HBV group in our findings could indeed be explained by the higher incidence of hypertensive disorders in this group that necessitated active management by induction, which could be associated with lower birthweights than the HBV-positive group.

The relationship between chronic HBV carrier status and the entire spectrum of hypertensive disorders in pregnancy continues to be controversial in the literature. Obviously, almost all available data in the literature including the current cohort were retrospective observational and epidemiological studies. Many of the studies aimed at reporting an adverse pregnancy outcome in chronic HBV carriers in general, often in communities where the prevalence of HBV-positive status was low, often less than 1% [14, 15]. However, HBV antigen carrier status in such low-prevalence areas might also be associated with lower socioeconomic status, drug addiction, other major medical conditions, or other high-risk factors that could account for adverse pregnancy outcome [16]. For instance, studies performed in low-HBV-burden settings as in the United States showed that most chronic carriers were Asians and/or foreign-born people [17] or those who had other high‐risk factors such as smoking, alcohol, and substance use and coinfection with HCV [18]. These studies would report an association of HBV carrier status with various perinatal adverse outcomes, often confounded by risk factors from social deprivation in these communities. Conversely, other large-scale studies from endemic areas would target towards assessing the correlation of HBV with major pregnancy complications such as preterm labour [19, 20] or gestational diabetes mellitus [21, 22], but often failed to address the issue of hypertensive disorders altogether.

Most of the studies that targeted at the association between HBV and hypertensive disorders in pregnancy or had at least examined this relationship specifically were able to show either a protective or neutral effect of positive HBV status. So far, large-scale population cohorts that showed a neutral association included many ethnic areas such as Thailand [7], North America [17, 18], Sweden [23], Israel [24], and China [3], with prevalence rates in these populations varying from 0.27 to 5.6%. Interestingly, the Swedish national study examined the pregnancy outcome of around 3000 women with HBV and 2000 with HCV and showed that maternal HCV was associated with a decreased risk of preeclampsia with an adjusted relative risk of 0.39 but not for HBV (HBV prevalence around 0.27%) [23]. On the other hand, studies that have reported a negative association between HBV and hypertensive disorders were exclusively large hospital-based cohort studies with the total number of women exceeding 10000 in Chinese populations with HBV prevalence rates of 10% or more [9–11]. A recent meta-analysis up to 2016 that included three observational cohorts and eight case-control studies with a total of 11566 preeclampsia patients also confirmed a significant negative association between chronic HBV and preeclampsia with OD 0.77 (95% confidence interval 0.65 to 0.90) [12]. Nevertheless, heterogeneity was inevitably present within these studies, as the definitions and classifications for hypertensive disorders would vary between different cohorts, while some studies only focused on preeclampsia and would exclude nonproteinuric gestational hypertensive disorders. In addition, the data included in this meta-analysis were based on the several large Chinese cohorts with high HBV prevalence rates, so that there is doubt whether the results could be generalized to other ethnic groups with lower prevalence rates.

Surprisingly, there were also isolated reports that showed a positive association with HBV aggravating the development of hypertensive disorders. A case-controlled study from Wuhan, China, showed an increase in the risk of pregnancy-induced hypertension (OR 2.20) in a group of 1728 HBV-positive women. The viral load in the second trimester in this cohort was reported to be relatively high in over 20% of them, which might indicate more active HBV infection in some of the women [6]. In another Sudanese cohort, where the prevalence of chronic HBV was quoted as 5.8%, a paradoxical increase in the incidence of hypertensive disorders was reported [5]. Remarkably, all studies that were able to identify a positive association were case-controlled studies rather than hospital-based or population-based cohorts, so that such findings were less likely to be generalizable. The key findings of major studies published within the past 20 years are summarized in chronological order.

The precise physiological mechanism for an association between chronic HBV and hypertensive disorders in pregnancy is still unclear. The immune response in chronic HBV infection is compatible with maternal immune tolerance which is thought to be similar to and extend to the fetus [2]. There was also mounting evidence that one of the critical pathophysiological mechanisms of preeclampsia could be the excessive activations of maternal innate and adaptive immune responses [25, 26]. The immune responses in preeclampsia, including a shift in favour of Thi cellular immune activity, activation of CD4+ and CD8+ nuclear cells, and increased systemic inflammation with the production of proinflammatory cytokines such as TNF- alpha [25], resembled the immune clearance phase targeted at the rejection of the conceptus. On the other hand, HBV replication was known to be able to induce virus-specific T-cell tolerance or T-cell depletion, causing relatively impaired adaptive immunity in those with chronic HBV infection [27, 28]. Indeed, such a mechanism of compromised immunity has been used to explain the unexpectedly lower incidence of Sjogren's syndrome, type-1 diabetes mellitus, and systemic lupus erythematosus had been found in HBV-infected patients as compared to non-HBV-infected controls [29]. Indeed, our data also showed a lower incidence of thyroid disorders in the HBV women as compared to the non-HBV group. As a predominant proportion of active thyroid disorders complicating pregnancy in our cohort were Graves's disease or autoimmune thyroiditis, the negative association could probably be explained by a similar mechanism [30]. In addition, as the immunological responses to preeclampsia would be expected to be much more prominent than those of milder gestational hypertension, we would explain our observation that the protective effect of HBV was more robust against preeclampsia as compared to gestational hypertension in our cohort. While the innate and adaptive immune responses to HBV varied with the infection time and the different evolutionary stages of chronic viral infection [31, 32], the precise interactions with the pathophysiological mechanisms to preeclampsia would also vary in the individual woman. The complex and variable responses between the virological and immunological responses to HBV and to preeclampsia could be one of the explanations to the conflicting results between their association. With the recent developments in the use of antenatal antiviral treatment to reduce maternal-fetal transmission of HBV infection based on maternal HBV DNA titres [33, 34], it would be interesting to evaluate whether different levels of HBV DNA would affect the association between HBV infection and hypertensive disorders that we have observed.

There are currently many different protocols employed for screening and prediction of preeclampsia. Apart from biochemical markers and maternal uterine artery Doppler measurements [35, 36], some still utilize positive risk factors such as maternal age, parity, obesity, multiple pregnancy, history of chronic hypertension, or renal diseases [37, 38]. While HBV status probably had only a modest effect as a negative predictor for preeclampsia, the inclusion of hepatitis B status as a protective factor in such preeclampsia screening protocols could be explored in future research. Nevertheless, the precise value of adding in such a parameter into the prediction model and whether other factors such as prevalence of HBV and ethnicity should also be considered would require further large-scale prospective studies.

The strengths of this study included the very large cohort collected over twenty years with a homogeneous population in a single centre. The limitations include the fact that the study is a retrospective cohort and that assessment of HVB DNA and the use of antenatal viral therapy are not yet part of our routine protocol during the collection of the cohort, so that we have no data on HBV DNA titres or other virological parameters. Finally, this cohort was based on a Chinese obstetric population with a high prevalence of HBV carrier status, yet a relatively low incidence of gestational hypertensive disorders and preeclampsia of around 4-5% and 2%, respectively, so that the findings might not be generalizable to other racial populations before further validation.

## 5. Conclusions

Our very large cohort continued to suggest a consistent and negative association between chronic hepatitis B carrier status and hypertensive disorders in pregnancy in a Chinese population. The protective effect of HBV against preeclampsia was apparently persistent despite a falling prevalence in HBV, though the HBV prevalence of 5-6% is still much higher than that of many nonendemic countries. Further research into the basic hepatic immunological mechanisms is warranted to explain our present epidemiological observation.

## Figures and Tables

**Table 1:** Prevalence of hepatitis B surface antigen carrier status, 2000–2019.

Year	Total deliveries	Number with HBV-positive status (%)
2000	3850	386 (10%)
2001	3522	370 (10.5%)
2002	3806	393 (10.3%)
2003	3787	384 (10.1%)
2004	4558	463 (10.2%)
2005	5078	527 (10.4%)
2006	4244	507 (11.9%)
2007	4754	539 (11.3%)
2008	5234	554 (10.6%)
2009	4951	551 (11.1%)
2010	5315	608 (11.4%)
2011	5552	616 (11.1%)
2012	4968	460 (9.3%)
2013	4079	337 (8.3%)
2014	4429	356 (8.0%)
2015	4317	339 (7.8%)
2016	4258	308 (7.2%)
2017	4107	273 (6.6%)
2018	3640	247 (6.8%)
2019	3440	184 (5.3%)

**Table 2:** Comparison of pregnancy complications and outcome between chronic hepatitis B carriers and noncarriers.

	HBV negative (*n* = 79487) (90.4%)	HBV positive (*n* = 8402) (9.56%)	*p* value
Ethnicity			
Chinese	78457 (98.7%)	8235 (98%)	<0.001
Non-Chinese	1030 (1.3%)	167 (2%)	
Parity			
Nulliparous	39178 (49.3%)	4082 (48.5%)	0.17
Multiparous	40309 (50.7%)	4320 (51.5%)	
Advanced maternal age	21670 (27.2%)	2296 (27.3%)	0.82
Antenatal anemia	4926 (6.19%)	521 (6.2%)	0.98
Cardiac diseases	843 (1.06%)	80 (9.5%)	0.35
Diabetes mellitus			
Preexisting	130 (1.6%)	8 (0.09%)	0.14
Gestational diabetes	6877 (8.65%)	763 (9.08%)	
Thyroid disorders	2125 (2.67%)	191 (2.27%)	0.029
Renal diseases	207 (0.26%)	24 (0.28%)	0.67
Liver diseases			
Non-hepatitis B related	95 (0.12%)	7 (0.08%)	<0.001
Hepatitis B related^*∗*^	—	44 (0.5%)	
Respiratory diseases	910 (1.14%)	77 (0.91%)	0.06
Epilepsy and other neurological disorders	156 (0.19%)	11 (0.13%)	0.19
Psychiatric disorders	1897 (2.38%)	203 (2.41%)	0.85
Immunological disorders	104 (0.13%)	14 (0.16%)	0.39
Antepartum haemorrhage	1769 (2.22%)	190 (2.26%)	0.83
Surgical disorders	144 (0.18%)	13 (0.15%)	0.58
Hypertensive disorders	3566 (4.48%)	260 (3.09%)	<0.001
Smoking	1604 (2.02%)	153 (1.82%)	0.22
Pregnancy outcome			
Livebirth	79147 (99.6%)	8373 (99.67%)	0.53
Stillbirth	235 (0.29%)	18 (0.2%)	
Neonatal death	105 (0.11%)	11 (0.13%)	
Obesity (BMI >25 kg/m^2^)	18362 (23.1%)	1991 (23.7%)	0.18
Preterm delivery (<37 weeks)	5299 (6.67%)	522 (6,21%)	0.12
Induction of labour	10562 (13.3%)	938 (11.1%)	<0.001
Mode of delivery			
Vaginal	56552 (71.2%)	5992 (71.3%)	0.11
Instrumental	5913 (7.4%)	639 (7.6%)	
Caesarean section	17022 (21.4%)	1771 (21.1%)	
5 min Apgar score <7	428 (0.54%)	54 (0.64%)	0.21
Small for gestational age	5356 (6.73%)	509 (6.05%)	0.018
Postpartum haemorrhage	4227 (5.31%)	452 (5.37%)	0.81

**Table 3:** Incidence of hypertensive complications in pregnancy.

	Total (*n* = 87889)			2000–2004 (*n* = 19523)			2005–2009 (*n* = 24261)			2010–2014 (*n* = 24343)			2015–2019 (*n* = 19763)		
	HBV −ve (*n* = 79487)	HBV +ve (*n* = 8402) (9.56%)	*p* value	HBV −ve (*n* = 17527)	HBV +ve (*n* = 1996) (10.2%)	*p* value	HBV −ve (*n* = 21583)	HBV +ve (*n* = 2678) (11.05%)	*p* value	HBV −ve (*n* = 21966)	HBV +ve (*n* = 2377) (9.7%)	*p* value	HBV −ve (*n* = 18411)	HBV +ve (*n* = 1351) (6.8%)	*p* value
Gestational hypertension^a^	2366 (2.97%)	185 (2.20%)	<0.001	524 (2.98%)	34 (1.70%)	<0.01	607 (2.81%)	57 (2.12%)	0.041	683 (3.11%)	63 (2.65%)	0.21	552 (2.99%)	31 (2.29%)	0.14
PET without chronic HT^b^	1103 (1.25%)	66 (0.78%)	<0.001	195 (1.11%)	12 (0.60%)	0.047	282 (1.30%)	19 (0.71%)	0.043	319 (1.45%)	23 (0.97%)	0.07	307 (1.67%)	12 (0.89%)	0.038
Chronic HT	168 (0.21%)	19 (0.22%)	0.87	13 (0.07%)	1 (0.05%)	0.94	34 (0.15%)	4 (0.15%)	0.87	54 (0.24%)	6 (0.25%)	0.85	67 (0.36%)	8 (0.59%)	0.27
PET superimposed on chronic HT^c^	60 (0.07%)	4 (0.04%)	0.24	9 (0.05%)	0	—	17 (0.08%)	3 (0.11%)	0.76	14 (0.06%)	0	—	20 (0.11%)	1 (0.07%)	0.95
Eclampsia^d^	37 (0.04%)	5 (0.06%)	0.68	12 (0.07%)	2 (0.1%)	0.94	12 (0.06%)	3 (0.11%)	0.95	8 (0.03%)	0	—	5 (0.03%)	0	—
All PET^(e=b+c+d)^	1200 (1.50%)	75 (0.89%)	<0.001	216 (1.23%)	14 (0.70%)	0.037	311 (1.44%)	25 (0.93%)	0.034	341 (1.55%)	23 (0.97%)	0.016	332 (1.80%)	13 (0.96%)	0.023
Total hypertensive disorders^(a+e)^	3566 (4.48%)	260 (3.09%)	<0.001	740 (4.22%)	48 (2.40%)	<0.001	918 (4.25%)	82 (3.06%)	0.001	1024 (4.66%)	86 (3.62%)	0.02	884 (4.80%)	44 (3.26%)	0.01

PET = preeclampsia, HT = hypertension.

**Table 4:** Major risk factors for women with or without preeclampsia and all pregnancy hypertensive disorders.

	PET (*n* = 1275) (1.45%)	No PET (*n* = 86614) (98.55%)	*p* value	All gestational hypertensive disorders (*n* = 3826) (%)	No gestational hypertension (*n* = 84063) (%)	*p* value
Chinese ethnicity	1263 (99%)	85429 (98.6%)	0.192	3784 (98.9%)	82895 (98.6%)	0.11
Parity						
Nulliparous	691 (54.2%)	42569 (49.1)	<0.001	2071 (54.1%)	41189 (49%)	<0.001
Multiparous	584 (45.8%)	44045 (50.9%)		1755 (45.9%)	42875 (51%)	

Advanced maternal age (>35)	414 (32.5%)	23499 (27.1%)	<0.001	1169 (30.6%)	22744 (27.1%)	<0.001
Multiple pregnancies	46 (3.6%)	1437 (1.65%)	<0.001	126 (3.29%)	1357 (1.61%)	<0.001
Obesity (body mass index >25 kg/m^2^)	360 (28.2%)	19962 (23%)	<0.001	950 (24.8%)	19372 (23%)	0.01
Smoking	21 (1.64%)	1736 (2%)	0.36	71 (1.85%)	1686 (2.01%)	0.52
Medical disorders^*∗*^	45 (3.53%)	942 (1.08%)	<0.001	76 (1.98%)	911 (1.08%)	0.001
Hepatitis B carrier status	75 (5.88%)	8327 (9.61%)	<0.001	260 (6.79%)	8142 (9.68%)	<0.001

^*∗*^All significant medical disorders that could predispose to gestational hypertension/preeclampsia, such as chronic hypertension, renal disorders, preexisting and gestational diabetes mellitus, and autoimmune disorders.

**Table 5:** Logistic regression of risk factors associated with the presence or absence of gestational hypertensive disorders and preeclampsia.

Risk factors	B	SE	Wald	Significance	Odds ratio	95% confidence interval
For all gestational hypertension						
Advanced maternal age	0.223	0.037	36.17	<0.001	1.25	1.16 to 1.35
Multiparity	−0.238	0.034	48.14	<0.001	0.78	0.74 to 0.84
Multiple pregnancies	0.678	0.095	50.89	<0.001	1.97	1.63 to 2.37
Obesity	0.098	0.038	6.36	0.012	1.10	1.02 to 1.19
Medical disorders	0.560	0.12	21.47	0.001	1.75	1.38 to 2.22
Hepatitis B carrier status	−0.385	0.065	34.72	0.001	0.68	0.60 to 0.77

For preeclampsia						
Advanced maternal age	0.307	0.062	24.24	<0.001	1.36	1.20 to 1.53
Multiparity	−0.239	0.059	16.57	<0.001	0.79	0.70 to 0.88
Multiple pregnancies	0.735	0.153	22.95	<0.001	2.09	1.54 to 2.81
Obesity	0.268	0.063	18.26	<0.001	1.31	1.16 to 1.48
Medical disorders	1.141	0.156	53.55	<0.001	3.13	2.31 to 4.25
Hepatitis B carrier status	−0.529	0.120	19.54	0.001	0.59	0.47 to 0.75

## Data Availability

The supporting database was derived from and under the ownership of the Hospital Authority (Hong Kong) Clinical Management System databases. The supporting data, in an anonymous format, are available from the corresponding author upon request.

## References

[1] Liu J., Liang W., Jing W., Liu M. (2019). Countdown to 2030: eliminating hepatitis B disease, China. *Bulletin of the World Health Organization*.

[2] Lao T. T. (2020). Hepatitis B chronic carrier status and pregnancy outcome: an obstetric perspective. *Best Practice & Research Clinical Obstetrics & Gynaecology*.

[3] Tan J., Liu X., Mao X. (2016). HBsAg positivity during pregnancy and adverse maternal outcomes: a retrospective cohort analysis. *Journal of Viral Hepatitis*.

[4] Zhao Y., Chen Y.-L., Song H.-Q. (2020). Effects of maternal hepatitis B surface antigen positive status on pregnancy outcomes: a retrospective study in Xiamen, China 2011-2018. *PLoS One*.

[5] Ahmed M. A., Shrif M. E., Rayis D. A., Nasr A. M., Adam I. (2018). Hepatitis B infection and pre-eclampsia among pregnant Sudanese women. *Virology Journal*.

[6] Wan Z., Zhou A., Zhu H. (2018). Maternal hepatitis B virus infection and pregnancy outcome: a hospital-based case-control study in Wuhan, China. *Journal of Clinical Gastroenterology*.

[7] Sirilert S., Traisrisilp K., Sirivatanapa P., Tongsong T. (2014). Pregnancy outcomes among chronic carriers of hepatitis B virus. *International Journal of Gynecology & Obstetrics*.

[8] Huang X., Tan H., Li X., Zhou S., Wen S. W., Luo M. (2014). Maternal chronic HBV infection would not increase the risk of pregnancy induced hypertension - results from pregnancy cohort in Luiyang rural China. *PLoS One*.

[9] Lao T. T., Chan B. C. P., Leung W. C., Ho L. F., Tse K. Y. (2007). Maternal hepatitis B infection and gestational diabetes mellitus. *Journal of Hepatology*.

[10] Lao T. T., Sahota D. S., Cheng Y. K. Y., Law L. W., Leung T. Y. (2013). Maternal hepatitis B surface antigen status and incidence of pre-eclampsia. *Journal of Viral Hepatitis*.

[11] To W. W. K., Cheung W., Mok K. M. (2003). Hepatitis B surface antigen carrier status and its correlation to gestational hypertension. *The Australian and New Zealand Journal of Obstetrics and Gynaecology*.

[12] Huang Q. T., Chen J. H., Zhong M., Hang L. L., Wei S. S., Yu Y. H. (2016). Chronic hepatitis B infection is associated with decreased risk of pre-eclampsia: a meta-analysis of observational studies. *Cellular Physiology and Biochemistry*.

[13] Lao T. T., Sahota D. S., Law L. W., Cheng Y. K. Y., Leung T. Y. (2014). Age-specific prevalence of hepatitis B virus infection in young pregnant women, Hong Kong Special Administrative Region of China. *Bulletin of the World Health Organization*.

[14] Pastorek J. G., Miller J. M., Summers P. R. (1988). The effect of hepatitis B antigenemia on pregnancy outcome. *American Journal of Obstetrics and Gynecology*.

[15] Lobstein S., Faber R., Tillmann H. L. (2011). Prevalence of hepatitis B among pregnant women and its impact on pregnancy and newborn complications in a tertiary hospital in the eastern part of Germany. *Digestion*.

[16] Wong S., Chan L. Y., Yu V., Ho L. C. (1999). Hepatitis B carrier and perinatal outcome in singleton pregnancy. *American Journal of Perinatology*.

[17] Bajema K. L., Karita H. C. S., Tenforde M. W., Hawes S. E., Heffron R. (2018). Maternal hepatitis B infection and pregnancy outcome in the United States: a population based cohort study. *Open Forum Infectious Diseases*.

[18] Reddick K. L., Jhaveri R., Gandhi M., James A. H., Swamy G. K. (2011). Pregnancy outcomes associated with viral hepatitis. *Journal of Viral Hepatitis*.

[19] Cui A. M., Shao J. G., Li H. B. (2017). Association of chronic hepatitis B infection with preterm birth: our experience and meta-analysis. *Journal of Perinatal Medicine*.

[20] Ma Z., Sun D., Li C., Ying J., Yan Y. (2018). Chronic hepatitis B virus infection and preterm labor(birth) in pregnant women—an updated systematic review and meta-analysis. *Journal of Medical Virology*.

[21] Lao T. T., Chung M. K., Cheung T. K. W., Law L. W. (2016). Antenatal hepatitis B and increased risk of gestational diabetes mellitus—implications for obstetric care. *Journal of Infection*.

[22] Peng S., Wan Z., Lin X., Li X., Du Y. (2019). Maternal hepatitis B surface antigen carrier status increased the incidence of gestational diabetes mellitus. *BMC Infectious Diseases*.

[23] Stokkeland K., Ludvigsson J. F., Hultcrantz R. (2017). Pregnancy outcome in more than 5000 births to women with viral hepatitis: a population-based cohort study in Sweden. *European Journal of Epidemiology*.

[24] Safir A., Levy A., Sikuler E., Sheiner E. (2010). Maternal hepatitis B virus or hepatitis C virus carrier status as an independent risk factor for adverse perinatal outcome. *Liver International*.

[25] Laresgoiti-Servitje E., Gomez-Lopez N., Olson D. M. (2010). An immunological insight into the origins of pre-eclampsia. *Human Reproduction Update*.

[26] Ahn H., Park J., Gilman-Sachs A., Kwak Kim J. (2011). Immunologic characteristics of preeclampsia. *American Journal of Reproductive Immunology*.

[27] Bertoletti A., Gehring A. J. (2006). The immune response during hepatitis B virus infection. *Journal of General Virology*.

[28] Stoop J. N., van der Molen R. G., Baan C. C., van der Lann L. J., Kuipers J. G., Janssen H. L. (2005). Regulatory T cells contribute to the impaired immune response in patients with chronic hepatitis B virus infection. *Hepatology*.

[29] Ram M., Anaya J. M., Barzilai O. (2008). The putative protective role of hepatits B virus (HBV) infection from autoimmune disorders. *Autoimmunity Reviews*.

[30] Cui W., Deng B., Wang W., Liu P. (2016). Graves’ hyperthyroidism accompanied with acute hepatitis B virus infection: an extrahepatic manifestation?. *Virology Journal*.

[31] Balmasova I. P., Yushchuk N. D., Mynbaev O. A. (2014). Immunopathogenesis of chronic hepatitis B. *World Journal of Gastroenterology*.

[32] Park J. J., Wong D. K., Wahed A. S. (2016). HBV –specific and global T-cell dysfunction in chronic hepatitis B. *Gastroeneterology*.

[33] Pan C. Q., Duan Z., Dai E. (2016). Tenofovir to prevent hepatitis B transmission in mothers with high viral load. *The New England Journal*.

[34] Li W., Jia L., Zhao X., Wu X., Tang H. (2018). Efficacy and safety of tenofovir in preventing mother-to-infant transmission of hepatitis B virus: a meta-analysis based on 6 studies from China and 3 studies from other countries. *BMC Gastroenterology*.

[35] Poon L. C., Shennan A., Hyett J. A. (2019). The International Federation of Gynecology and Obstetrics (FIGO) Initiative on preeclampsia (PE): a pragmatic guide for first trimester screening and prevention. *International Journal of Gynaecology & Obstetrics*.

[36] Taca A., Romero R., Benshalom-Tirosh N. (2019). The prediction of early preeclampsia; results from a longitudinal proteomics study. *PLoS One*.

[37] Park H. J., Shim S. S., Cha D. H. (2015). Combined screening for early detection of preeclampsia. *International Journal of Molecular Sciences*.

[38] Al-Rubaie Z. T. A., Hudson H. M., Jenkins G. (2020). Prediction of preeclampsia in nulliparous women using routinely collected maternal characteristics: a model development and validation study. *BMC Pregnancy and Childbirth*.

